# In vivo labeling reveals that degranulation is increased under supraphysiological TCR stimulation, but not infection, in CD8^+^ T cells from old mice

**DOI:** 10.1007/s11357-025-01723-5

**Published:** 2025-06-06

**Authors:** Korbyn J. V. Dahlquist, Emma M. Dehm, Declan M. Smith, Erin D. Lucas, Mark Pierson, Sara E. Hamilton, Christina D. Camell

**Affiliations:** 1 Biochemistry, Molecular Biology, and Biophysics Graduate Program, Department of Biochemistry, Molecular Biology, and Biophysics, University of Minnesota, Minneapolis, MN USA; 2 Masonic Institute On the Biology of Aging and Metabolism, Department of Biochemistry, Molecular Biology, and Biophysics, University of Minnesota, Minneapolis, MN USA; 3 Center for Immunology, University of Minnesota, Minneapolis, MN USA; 4 Microbiology, Immunology, and Cancer Biology Graduate Program, University of Minnesota Medical School, Minneapolis, MN USA; 5 Department of Laboratory Medicine and Pathology, University of Minnesota, Minneapolis, MN USA

**Keywords:** CD8+ T cells, Cytotoxicity, Degranulation, Aging, Infection

## Abstract

CD8^+^ T cells exhibit distinct changes with aging, including a diminished naïve cell pool, an expansion of memory and exhausted cells, and altered effector molecule production, altogether leading to increased susceptibility to infection. They have reduced cytotoxicity in vivo, but increased granule content and faster cytotoxic kinetics to target cells in vitro*.* Whether CD8^+^ T cells from old mice degranulate when activated in vivo*,* within the aged environment, is unknown. This study investigates in vitro and in vivo degranulation of CD8^+^ T cells from young and old mice during supraphysiological aCD3 stimulation and two types of infection. Actively degranulating CD8^+^ CD44^+^ T cells were identified by positive labeling after a two-hour exposure to granule-specific fluorescent antibodies (CD107a and CD107b). Surprisingly, CD8^+^ T cells from old mice challenged with supraphysiological TCR-specific stimulation exhibited higher levels of degranulation as compared to their young counterparts. This effect is more prominent in vitro and can be partially explained by the age-specific increase in CD8^+^ CD44^+^ CD62L^−^ cells. However, during microbial exposure or LCMV Armstrong infection, we show that CD8^+^ CD44^+^ and antigen-specific T cells from old mice have reduced degranulation, consistent with the diminished cytotoxic capacity. These data highlight the preserved intrinsic cytotoxic capacity of memory CD8^+^ T cells from old mice and suggest that the aged microenvironment and type of stimulation are contributing factors to the lower degranulation and cytotoxic capacity of these cells. This provides insight into the potential of increasing T cell activation to improve vaccine approaches in the elderly.

**Supplementary Information:**

The online version contains supplementary material available at 10.1007/s11357-025-01723-5.

## Introduction

The ability of the immune system to respond to infectious challenges declines with increasing age, leading to increased hospitalization, increased mortality, and chronic repercussions [1, 2]. In older individuals, there is a systemic increase in sterile inflammation and expanded dysfunctional immune cell subsets that are hyper- and hypo-responsive depending on the stimuli or cellular context [3–6]. These major age-associated alterations of the immune system are collectively referred to as immunosenescence. One of the earliest and most apparent age-related changes in the immune system is the involution of the thymus, with reduced thymus size and structure resulting in a diminished naïve T cell pool [7, 8]. Due, in part, to reduced naïve T cell numbers, the antigen-experienced CD8^+^ T cell repertoire generated after antigen exposure is constricted [9]. CD8^+^ T cell functionality declines with age and is associated with the emergence and expansion of distinct age-related T cell subsets, including virtual memory cells, Granzyme K^+^ PD1^+^ terminally exhausted cells, and senescent-like cells [10–14]. Altogether, these alterations in CD8^+^ T cell function drive aging and lead to reduced ability to manifest sterile immunity; however, the exact molecular mechanisms leading to changes in T cell function are multifactorial and incompletely understood [13, 15–20].

CD8^+^ T cell cytotoxicity is dependent on intrinsic cell function and extrinsic, environmental factors, including priming from antigen-presenting cells (APCs) and inflammatory signaling via cytokines (signal 3) [21, 22]. In infection models in aged mice, CD8^+^ T cells exhibit reduced production of IFN-γ, TNF-α, and IL-2 effector cytokines and reduced cytotoxicity [6, 12, 23, 24]. Primarily, these studies examine cytotoxicity in vivo and cannot distinguish between extrinsic and intrinsic dysfunction. The literature that reveals intrinsic alterations of CD8^+^ T cell cytotoxic machinery with age, Zöphel et al., showed that CD8^+^ T cells from aged individuals have increased cytotoxic granule levels of granzyme B (GzmB) and perforin in vitro. In addition to increased granule content, aged CD8^+^ T cells have faster killing kinetics and are more efficient killers than CD8^+^ T cells from young mice [25]. Together, this work builds on studies by Saxena & Adler, which showed that aged CD8^+^ had enhanced cytotoxic activity against some target cell lines, but not others, in vitro compared to cells from young mice [26, 27]. The reasons for the discrepancy are not entirely clear but may be explained by evaluating T cells in vitro and in vivo. These current in vivo studies are also limited by an incomplete understanding of the cytotoxic process.

Cytotoxic granules are secretory lysosomes containing perforin and granzymes; additionally, they express CD107a (Lamp-1) and CD107b (Lamp-2) lysosomal proteins on their inner leaflet. Upon formation of the immunological synapse, when CD8^+^ T cells recognize and bind to target cells, cytotoxic granules migrate to the cell surface, where they fuse and degranulate (or exocytose), releasing their contents into the synaptic cleft to lyse target cells [28]. Upon degranulation, CD107a and CD107b are transiently expressed on the cell surface. Here, we employed a unique technique established in Yuzefpolskiy et al. to detect degranulation in old mice both in vitro and in vivo [29]. The data presented here details the use of in vivo CD107a and CD107b labeling to define CD8^+^ T cell degranulation from old mice. This work allows for the dissection of CD8^+^ T cell-extrinsic and intrinsic cytotoxic capacity by assessing direct degranulation. Here, we reveal a significant increase in CD8^+^ CD44^+^ T cells that express high levels of CD107a and CD107b after aCD3 stimulation in old mice compared to young mice in vitro and in vivo. We find that the expression of CD107a/CD107b is highest in CD8^+^ CD44^+^ CD62L^−^ effector T cells (Tems). Conversely, we showed that in a physiological infection (cohousing with a petstore mouse; CoH) CD107a/CD107b is expressed in young but not old CD8^+^ CD44^+^ T cells. Understanding the underlying mechanism of altered cytotoxicity of aged CD8^+^ T cells will reveal new mechanistic targets to improve the CD8^+^ T cell response during infectious disease in aged individuals.

## Material and methods

### Animal care and ethics statement

All mice were housed in AALAC-approved specific pathogen-free (SPF) animal facilities in HEPA-filtered cages with free access to sterile water and normal chow diet (2018 Teklad Global 18% Protein Rodent Diets) and housed under 14/10 h light/dark cycles at the University of Minnesota. Wild-type (WT) C57BL/6 J mice were bred from our colony at the University of Minnesota, purchased from Jackson Laboratory (Bar Harbor, ME) or received from the Aging Rodent Colony at the National Institute of Aging (Baltimore, MD). We define young mice as 3–6 months of age and old mice as 18–24 months of age. The precise ages of the mice used can be found in the animal experiments section of the methods. Old mice with frailty, pathology, or tumors were excluded from the study. All experiments and animal use were conducted in compliance with the National Institute of Health Guide for the Care and Use of Laboratory Animals, were approved by the Institutional Animal Care and Use Committee at the University of Minnesota, and performed in accordance with the National Institutes of Health guidelines for ethical animal treatment. Petstore mouse cohousing (CoH) was conducted as described [6, 30].

### Intravascular stimulation and staining

In vivo stimulation was performed by retro-orbital intravenous injection (i.v.) of 0.2ug/g anti-CD3e (aCD3) functional grade monoclonal antibody (Invitrogen; 16–0031–86) for 1 h. After 1 h, intravascular labeling was performed by retro-orbital injection of 30ug PE CD107a (Biolegend; 121,612) and 30ug PE-CD107b (Biolegend; 108,506) or 30ug PE- Rat IgG2a, κ (Biolegend; 400,507) and 30ug Rat PE-IgG1, κ (Biolegend; 400,407) isotype controls (PE-Isotypes). Mice were euthanized 2 h after CD107a/CD107b labeling.

### LCMV-Armstrong infection

Young and old mice were infected with 2 × 10^5^ PFU of LCMV-Armstrong intraperitoneally (i.p.) in a 100 μL volume. For in vivo analysis, on day 5 of infection, intravascular labeling was performed by retro-orbital injection of 30ug PE CD107a (Biolegend; 121,612) and 30ug PE-CD107b (Biolegend; 108,506) or 30ug PE- Rat IgG2a, κ (Biolegend; 400,507) and 30ug Rat PE-IgG1, κ (Biolegend; 400,407) isotype controls (PE-Isotypes). Mice were euthanized 2 h after CD107a/CD107b labeling.

### Animal experiments

List of Animal experiments. Each experiment is listed with a title describing the purpose of the experiment. If experiments are pooled data, individual cohorts are described as parts.

#### Experiment 1- In vitro TCR stimulation and degranulation detection of splenocytes from young and old mice (three individual cohorts compiled)

- Part 1: WT-C57BL/6 J; *N = *3 young (3-months-old), *N = *3 old (21-months-old); Female mice.
- Part 2: WT-C57BL/6 J; *N = *3 young (3-months-old), *N = *3 old (21-months-old); Female mice.
- Part 3: WT-C57BL/6 J; *N = *2 young (2-months-old); Female mice.

#### Experiment 2- Young and old CD8.+ CD3 and TCRβ expression analysis and CD3 titration in vitro degranulation (two individual cohorts compiled)

- Part 1: WT-C57BL/6 J; *N = *3 young (2.5-months-old); *N = *3 old (22-months-old); Female mice.
- Part 2: WT-C57BL/6 J; *N = *4 young (2.5-months-old); *N = *4 old (22-months-old); Female mice.

#### Experiment 3- In vivo degranulation in young and old aCD3-treated mice (three individual cohorts compiled)

- Part 1: WT-C57BL/6 J; Young (5-months-old) and old (21–24-months-old): *N = *1 young Isotype + PE-Isotypes; *N = *1 young aCD3 + PE-Isotypes; *N = *3 young Isotype + PE-CD107a/CD107b; *N = *3 young aCD3 + PE-CD107a/CD107b, *N = *1 old Isotype + PE-Isotypes; *N = *1 old aCD3 + PE-Isotypes; *N = *3 old Isotype + PE-CD107a/CD107b; *N = *3 old aCD3 + PE-CD107a/CD107b; Female mice.
- Part 2: WT-C57BL/6 J; Young (5-months-old) and old (21–22-months-old): *N = *1 young aCD3 + PE-CD107a/CD107b, *N = *2 old aCD3 + PE-CD107a/CD107b; Female mice.
- Part 3: WT-C57BL/6 J; Young (3-months-old) and old (19-months-old): *N = *3 young aCD3 + PE-CD107a/CD107b, *N = *1 old aCD3 + PE-Isotypes, *N = *5 old aCD3 + PE-CD107a/b; Female mice.

#### Experiment 4- In vivo degranulation in young and old CoH mice (two individual cohorts compiled)

- Part 1: WT-C57BL/6 J; Young (3-months-old); old (21-months-old): *N = *1 young PE-Isotypes; *N = *2 young PE-CD107a/CD107b; *N = *2 old PE-CD107a/CD107b; Female mice.
- Part 2: WT-C57BL/6 J; Young (4-months-old); old (22-months-old): *N = *1 young PE-Isotypes; *N = *4 young PE-CD107a/CD107b; *N = *2 old PE-CD107a/CD107b; Female mice.

#### Experiment 5- In vivo degranulation in young and old LCMV-Armstrong infected mice

WT-C57BL/6 J; Young (5-months-old); old (21-months-old): *N = *1 young PE-Isotypes; *N = *8 young PE-CD107a/CD107b; *N = *1 old PE-Isotypes; *N = *7 old PE-CD107a/CD107b; Female mice.

### Immune cell isolation

Spleens were harvested and stored in RPMI supplemented with 10% FBS on ice before processing. Immune cells were isolated from whole spleens using mechanical digestion by mashing through 100 um filters. Red blood cells were lysed using ACK lysis buffer for 3 min at room temperature (25 °C), followed by a 40 µm filtration to remove cellular debris.

### Flow cytometry staining

For cell surface staining, splenocytes were plated into a 96-round-bottom plate. Cells were stained with fixable Ghost Dye Red 780 viability dye (Tonbo; 13–0865-T100) for 25 min at 4 °C. Cells were incubated with FcBlock and surface antibodies for 45 min at 4 °C. For nuclear staining, cells were fixed and permeabilized using the eBioscience FOXP3/Transcription factor staining buffer set (Invitrogen; 00–5521-00) according to the manufacturer’s instructions, followed by nuclear antibody staining for 45 min at 4 °C. Flow cytometry data were acquired on a FACSymphony A3 R6609 (BD Biosciences) using BD FACSDiva Software v 9.0 and analyzed using Flowjo software version 10.9. Gating strategies are provided in Supplementary Fig. 1a.

### Splenocyte stimulation

1ug aCD3e functional grade monoclonal antibody (Invitrogen;16–0031–86;) or Armenian hamster IgG isotype control (Invitrogen; 16–4888-81) was plate-bound in a 24 flat-bottom well plate for 24 h at 4 °C. To identify LCMV-specific cells, H2D^b^ gp33 monomer was tetramerized with streptavidin-APC and then included in the surface stain. Excess antibody was washed with cold PBS. 2 × 10^6^ splenocytes were seeded into the wells and media was supplemented with 10uL of PE CD107a (Biolegend; 121,612) and 10uL PE CD107b (Biolegend; 108,506) or 10uL PE- Rat IgG2a, k (Biolegend; 400,507) and 10uL Rat PE-IgG1, k (Biolegend; 400,407) isotype controls for 3 h. RPMI supplemented with 10% FBS, antibiotics and antimycotics (Gibco; 15,240,062), HEPES, Glutamax (Gibco; 35,050,061), and 2-mercaptoethanol was used as the stimulation media.

### Antibodies

**Table:** 

Fluorophore	Antigen	Company	Catalogue	Clone	dilution
BUV395	CD8a	BD Biosciences	563,786	53–6.7	1:200
BUV737	CD4	BD Biosciences	612,761	GK1.5	1:200
BV421	CD69	Biolegend	104,527	H1.2 F3	1:200
BV421	NK1.1	Biolegend	108,741	PK136	1:200
BV605	CD44	BD Biosciences	563,058	IM7	1:200
BV785	CD45	Biolegend	103,149	30-F11	1:200
PerCPCY5.5	CD62L	BD Biosciences	560,512	MEL-14	1:200
FITC	GzmB	Biolegend	515,403	GB11	1:10
APC	TCRβ	BD Biosciences	561,080	H57-597	1:200
PE	CD3	Invitrogen	11–0031–85	17 A2	1:200
Biotin	H2D^b^ gp33 monomer	NIH Tetramer Core Facility	LCMV gp33-41	KAVYNFATC	1:300
APC	Streptavidin	Thermo Fisher	S32362	NA	
PE	CD107a	Biolegend	121,612	ID4B	
PE	CD107b	Biolegend	108,506	M3/84	
PE	Rat IgG2a, k	Biolegend	400,507	RTK2758	
PE	Rat IgG1, k	Biolegend	400,407	RTK2071	

### Statistics and reproducibility

Statistical analysis was performed using GraphPad Prism version 10.3.1 (GraphPad Software Inc., USA). The type of statistical test run is listed in the figure legends. Statistical significance was defined as *p < *0.05 and defined with *p < 0.05; **p < 0.005; ***p < 0.001; ****p < 0.0001. No statistical methods were used to determine sample sizes, but our sample sizes are similar and based on previously published or performed experiments. Data was assumed to be normal. Continuous variables were expressed as Mean ± standard error of the mean (SEM). No randomization method was used to allocate animals to control or experimental groups. Data collection and analysis were not performed blindly to the conditions of the experiments.

## Results

### Memory CD8^+^ CD44^+^ T cells from old mice have increased degranulation in vitro

**Fig. 1:**
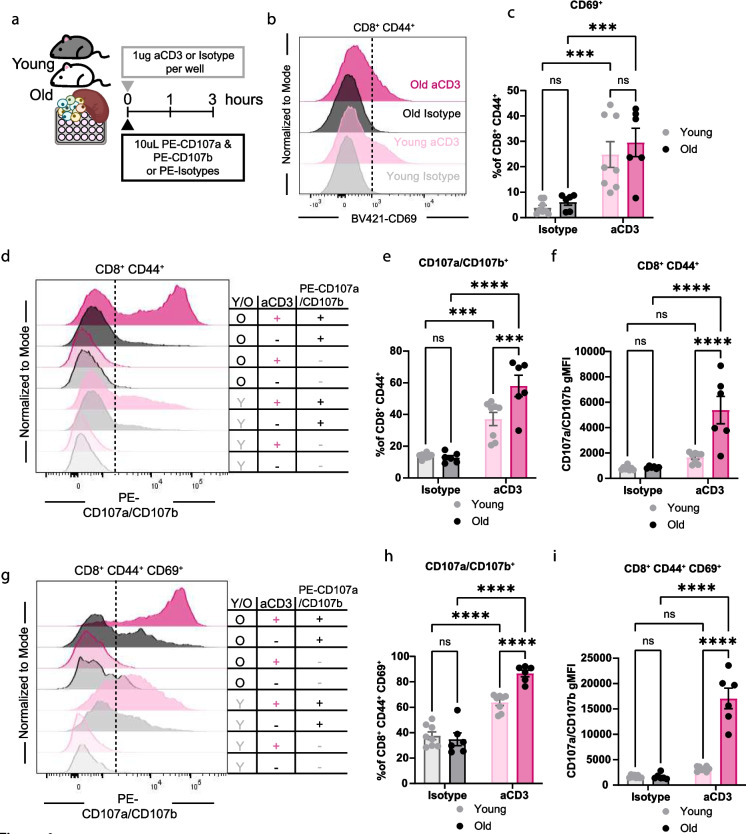
Memory CD8^+^ CD44^+^ T cells from old mice have increased degranulation in vitro. **a**. Diagram detailing experimental design. Stimulation of splenocytes from young and old mice with 1ug aCD3 and 10uL of PE-CD107a and 10uL PE-CD107b for 3 h in vitro. **b**. Concatenated histograms of CD69 expression on CD8^+^ CD44^+^ T cells. **c**. Quantification of CD69^+^ cells as a percentage of CD8^+^ CD44^+^ cells. **d**. Concatenated histograms of CD107a/CD107b expression on CD8^+^ CD44^+^ T cells. (Y) cells from young mice; (O) cells from old mice. (+) means addition of aCD3 or PE-CD107a/CD107b (-) addition of equivalent isotype controls. **e**. Quantification of CD107a/CD107b^+^ cells as a percentage of CD8^+^ CD44^+^ cells. **f**. Quantification of CD107a/CD107b gMFI on CD8^+^ CD44^+^ cells. **g**. Concatenated histograms of CD107a/CD107b expression on CD8^+^ CD44^+^ CD69^+^ T cells. **h**. Quantification of CD107a/CD107b^+^ cells as a percentage of CD8^+^ CD44^+^ CD69^+^ cells. **i**. Quantification of CD107a/CD107b gMFI on CD8^+^ CD44^+^ CD69^+^ cells. All data are presented as Means ± SEM. Statistical significance was determined with repeated measures two-way ANOVA with two-way ANOVA with Fisher’s Least Significant Difference multiple comparisons test. N and ages are listed in materials and methods under: **a-i** Experiment 1: young *N = *8; old *N = *6

To first evaluate the degranulation capacity of CD8^+^ T cells from young (3 months old) and old (21 months old) mice after T cell receptor (TCR) activation in vitro, splenocytes from young and old mice were isolated for analysis of degranulation. 2 × 10^6^ splenocytes were plated and stimulated with 1ug of plate-bound anti-CD3e (aCD3) or isotype control in the presence of 10ul of PE conjugated CD107a (PE-CD107a) and 10ul CD107b (PE-CD107b) or isotype controls (PE-isotype) for 3h (Fig. 1a). CD107a (Lamp-1) and CD107b (Lamp-2) are expressed on the inner leaflet of cytotoxic granule membranes; therefore, upon exocytosis of lytic granules, they are transiently expressed on the cell surface, allowing for antibody binding [31]. CD107a and CD107b surface expression have been correlated with the release of granzymes, perforin, and cytotoxic capabilities [28, 29, 32, 33].

CD8^+^ CD44^+^ memory cells are expanded with increasing age [34]. To define differences in the total memory pool between CD8^+^ T cells from young and old mice after aCD3 stimulation in vitro*,* we quantified CD44 expression (Supplementary Fig. 1a). We found that there is a significant expansion of CD8^+^ CD44^+^ with age, but not after 3 h of activation with aCD3 (Supplementary Fig. 2a). We next sought to determine if there were any differences in activation in memory CD8^+^ CD44^+^ T cells from young and old mice by evaluating CD69 expression, a marker of early T cell activation [35]. There was a significant increase in the frequency of CD8^+^ CD44^+^ CD69^+^ T cells with aCD3 stimulation, in cells from young and old mice, indicating robust activation with our model (Fig. 1b and c). Consistently, we also observed an increase in CD69^+^ cells as a frequency of naïve CD8^+^ CD44^−^ T cells with stimulation that wasn’t dependent on age (Supplementary Fig. 2b). Moreover, as aCD3 stimulation is not specific to CD8^+^ T cells, but rather all T cells, we explored the activation of CD4^+^ T cells and found an expansion of CD4^+^ CD44^+^ CD69^+^ in young aCD3-stimulated cells compared to young isotype and in old, aCD3-stimulated cells compared to old isotype mice (Supplementary Fig. 2c).

To quantify degranulation of memory CD8^+^ CD44^+^ T cells from young and old mice after TCR stimulation in vitro*,* we measured the frequency (CD107a/CD107b^+^) and expression (geometric mean fluorescence intensity; gMFI) of CD107a and CD107b. We found a significant increase in the frequency of CD8^+^ CD44^+^ CD107a/CD107b^+^ cells from young and old mice stimulated with aCD3 compared to isotype control-treated splenocytes (Fig. 1d and e). Interestingly, the frequency of CD8^+^ CD44^+^ CD107a/CD107b^+^ cells is significantly increased in old cells with aCD3 treatment as compared to young cells. Consistently, the gMFI of CD107a/CD107b on CD8^+^ CD44^+^ T cells was significantly increased in the old aCD3 stimulated cells compared to the young aCD3 treated cells (Fig. 1F). To confirm this phenotype and evaluate degranulation differences between activated and “non-activated” memory cells, we measured CD107a/CD107b on CD8^+^ CD44^+^ CD69^+^ and CD69^−^ T cells. Consistent with the total memory population, CD8^+^ CD44^+^ CD69^+^ frequency and gMFI of CD107a/CD107b is significantly expanded in old aCD3 compared to young aCD3 stimulated splenocytes (Fig. 1g-i). Additionally, CD8^+^ CD44^+^ CD69^−^ T cells from old aCD3 stimulated splenocytes have an increased frequency of CD107a/CD107b^+^ and gMFI compared to young aCD3 stimulated cells (Supplementary Fig. 2d-f). We found that the old aCD3 stimulated CD8^+^ CD44^+^ CD69^+^ CD107a/CD107b gMFI averaged 15,000, whereas the CD8^+^ CD44^+^ CD69^−^ was only 3000, consistent with the fact that activated cells degranulate more (Supplementary Fig. 2e and f).

We next sought to determine whether the increase in CD107a/CD107b expression between young and old CD8^+^ T cells after aCD3 stimulation was due to differential expression of CD3. We assessed CD3 and TCRβ surface expression on young and old CD8^+^ CD44^+^ T cells, either gated on CD3 or TCRβ (Supplementary Fig. 2g). We found no difference in CD3 gMFI, and significantly reduced TCRβ gMFI on old compared to young CD8^+^ CD44^+^ cells, gated on CD3^+^ and TCRβ^+^ cells, respectively (Supplementary Fig. 2h and i).

We next addressed whether CD107a/CD107b expression would be altered by different doses of aCD3 stimulation. We plated 0.001, 0.01, 0.1, 0.5, 1, 5, 10 ug of aCD3 and stimulated splenocytes for 3 h in the presence of PE-CD107a and PE-CD107b. At non-mitogenic doses (0.001, 0.01ug), we observed no surface expression of CD107a/CD107b (Supplementary Fig. 2j and k). However, at 0.1ug aCD3, CD8^+^ CD44^+^ cells from old mice showed a significant increase in the frequency of CD107a/CD107b^+^ compared to the young cells (Supplementary Fig. 2j). The increase in CD107a/CD107b^+^ cells in the old CD8^+^ CD44^+^ T cells was maintained at the 0.5, 1, 5, and 10ug aCD3 doses. Additionally, we observed a 2.7, 5.7, 4.7, 3.7, and 3.4-fold increase in CD107a/CD107b gMFI between the old and young cells at the 0.1, 0.5, 1, 5, and 10ug doses, respectively (Supplementary Fig. 2k). Together, these data indicate that old CD8^+^ CD44^+^ cells have increased degranulation in vitro that is not explained by differential CD3 expression.

### CD8^+^ CD44^+^ T cells from old mice have maintained degranulation in vivo

**Fig. 2:**
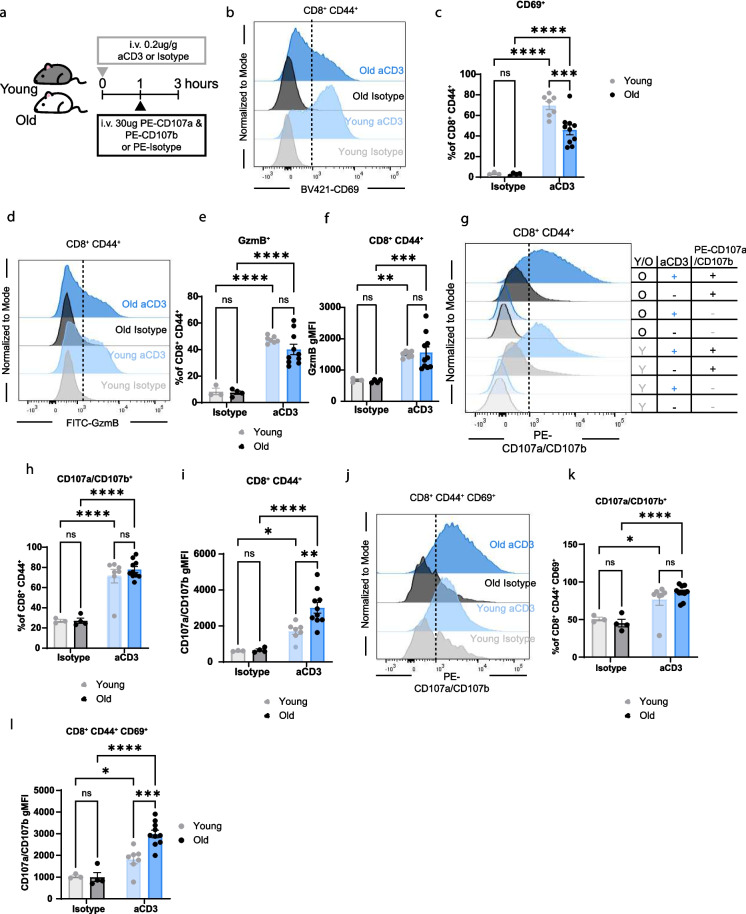
Memory CD8^+^ CD44^+^ T cells from old mice have maintained degranulation in vivo. **a**. Diagram detailing experimental design. Young and old mice were i.v. injected with 0.2ug aCD3 for 1 h. Mice were injected with 30ug PE-CD107a and 30ug PE-CD107b, after 2 additional hours mice were euthanized and tissues were processed for flow cytometry analysis. **b**. Concatenated histograms of CD69 expression on CD8^+^ CD44^+^ T cells. The dotted line represents the positive vs negative expression of CD69. **c**. Quantification of CD69^+^ cells as a percentage of CD8^+^ CD44^+^ cells. **d**. Concatenated histograms of GzmB expression on CD8^+^ CD44^+^ T cells. The dotted line represents the positive vs negative expression of GzmB. **e**. Quantification of GzmB^+^ cells as a percentage of CD8^+^ CD44^+^ cells. **f**. Quantification of GzmB gMFI on CD8^+^ CD44^+^ cells. **g**. Concatenated histograms of CD107a/CD107b expression on CD8^+^ CD44^+^ T cells. **h**. Quantification of CD107a/CD107b^+^ cells as a percentage of CD8^+^ CD44^+^ cells. The dotted line represents the positive vs negative expression of CD107a/CD107b. **i**. Quantification of CD107a/CD107b gMFI on CD8^+^ CD44^+^ cells. **j**. Concatenated histograms of CD107a/CD107b expression on CD8^+^ CD44^+^ CD69^+^ T cells. The dotted line represents the positive vs negative expression of CD107a/CD107b. **k**. Quantification of CD107a/CD107b^+^ cells as a percentage of CD8^+^ CD44^+^ CD69^+^ cells. **l**. Quantification of CD107a/CD107b gMFI on CD8^+^ CD44^+^ CD69^+^ cells. All data are presented as Means ± SEM. Statistical significance was determined with repeated measures two-way ANOVA with two-way ANOVA with Fisher’s Least Significant Difference multiple comparisons test. N and ages are listed in materials and methods under: **a-l** Experiment 3: young isotype *N = *3, young aCD3 *N = *7, old isotype *N = *4, old aCD3 *N = *10

Stimulation of cells in vitro does not fully recapitulate the complexity of biology in vivo. To understand CD8^+^ T cell biology, especially in aging, considering the increase in sterile, low-grade, chronic inflammation, in vivo models must be utilized. In vivo i.v. injection of fluorophore-conjugated CD107a and CD107b has been performed in young mice during infection. This work assessed the degranulation of memory precursor effector cells (MPEC) and short-lived effector cells (SLEC) and revealed the ability to detect active degranulation in vivo [29]. To quantify degranulation in vivo*,* we injected young and old mice with 0.2ug/g of aCD3 or isotype control antibody. After 1 h, they were i.v. injected with 30ug PE-CD107a and 30ug PE-CD107b or PE-isotype controls, and euthanasia occurred after an additional 2 h (Fig. 2a). We first determined whether in vivo TCR activation altered the frequency of CD8^+^ CD44^+^ T cells in young and old mice. The frequency of CD8^+^ CD44^+^ T cells was significantly increased with age but not altered after 3 h of aCD3 stimulation (Supplementary Fig. 3a). Next, we evaluated the activation of CD8^+^ CD44^+^ cells by quantifying the frequency of CD69^+^ and GzmB^+^ cells. Consistent with activation, CD8^+^ CD44^+^ CD69^+^ T cell frequency was increased with aCD3 stimulation but not further amplified by age (Fig. 2b and c). Additionally, the frequency of CD8^+^ CD44^+^ GzmB^+^ cells and GzmB gMFI was expanded with aCD3 stimulation but not age (Fig. 2d-f). CD8^+^ CD44^−^ CD69^+^ and GzmB^+^ frequencies and GzmB gMFI were also expanded with aCD3 stimulation but not age (Supplementary Fig. 3b-d). Together, these data indicate that 0.2ug/g of i.v. aCD3 activates CD8^+^ T cells in young and old mice to a comparable extent.

We next assessed the degranulation of memory CD8^+^ CD44^+^ T cells after aCD3 stimulation via expression of CD107a and CD107b using multi-parameter flow cytometry. We observed a significant increase in the frequency of memory CD8^+^ CD44^+^ CD107a/CD107b^+^ cells with aCD3 stimulation in cells from both young and old mice (Fig. 2g and h). This increase was also observed when quantifying CD107a/CD107b gMFI (Fig. 2i). Next, we measured the degranulation of activated (CD69^+^) and non-activated (CD69-) memory CD8^+^ CD44^+^ T cells. There was a significant expansion of CD107a/CD107b gMFI on CD8^+^ CD44^+^ CD69^+^ T cells in old aCD3 stimulated mice compared to young aCD3 stimulated mice, but not the frequency of CD107a/CD107b^+^ CD8^+^ CD44^+^ CD69^+^ T cells (Fig. 2j-l). When comparing degranulation of non-activated CD8^+^ CD44^+^ CD69^−^ T cells, there was a significant increase in degranulation with stimulation and age in CD107a/CD107b frequency or gMFI (Supplementary Fig. 3e-g).

This study is focused on the degranulation capacity of CD8^+^ T cells in vivo; however, aCD3 can also activate NKT cells, which are another subset of cells that exhibit strong cytotoxic function. Both NK and NKT cells are defined by their positive expression of NK1.1 and can be distinguished by their expression of CD3. However, we were unable to distinguish between these cell subsets as commercially available clones of CD3 overlap in their binding of CD3 and thus could not be labeled after in vivo stimulation with aCD3 monoclonal antibody. Therefore, we quantified CD107a/CD107b expression in both NK and NKT cells (NK1.1^+^). We observed no significant increases in the frequency of CD107a/CD107b^+^ NK1.1^+^ cells or CD107a/CD107b gMFI on NK1.1^+^ cells between young aCD3 and old aCD3 mice (Supplementary Fig. 3h-j). Together, these data show that CD8^+^ CD44^+^ T cells from old mice degranulate to a similar or greater extent than cells from young mice.

### CD8^+^ CD44^+^ CD62L^−^ T cells have the highest CD107a/CD107b expression in old mice

**Fig. 3:**
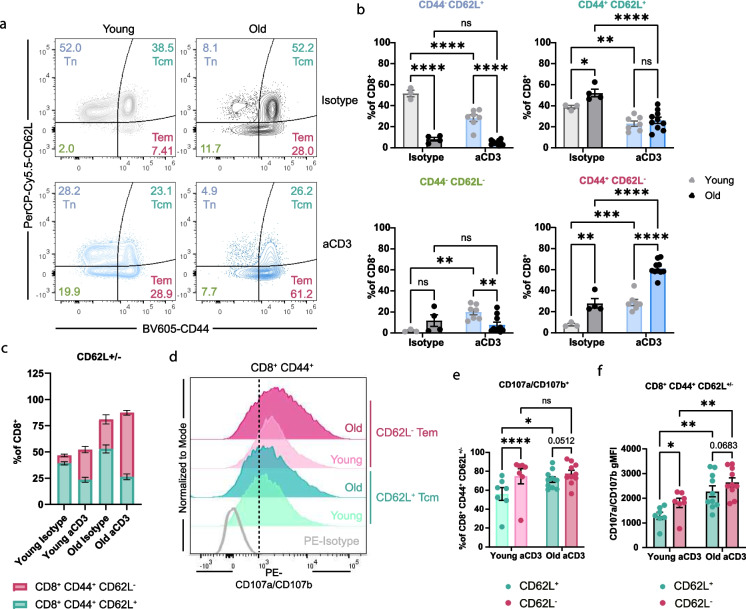
Tem CD8^+^ CD44^+^ CD62L^−^ T cells have the highest CD107a/CD107b expression in old mice. **a**. Concatenated contour plots of CD44 vs CD62L. Numbers are average frequencies. **b**. Quantification of memory cell populations as a frequency of CD8^+^ T cells. Each plot represents a different CD44 vs CD62L population. **c**. Quantification of CD44^+^ CD62L^+^ and CD44^+^ CD62L^−^ cells as a percentage of CD8^+^ cells. **d**. Concatenated histograms of CD107a/CD107b expression representing total counts in CD44^+^ CD62L^+^ and CD44^+^ CD62L^−^ memory populations. The dotted line represents the positive vs negative expression of CD107a/CD107b. **e**. Quantification of CD107a/CD107b^+^ cells as a percentage of CD8^+^ CD44^+^ CD62L^+^ or CD8^+^ CD44^+^ CD62L^−^ cells. **f**. Quantification of CD107a/CD107b gMFI on CD8^+^ CD44^+^ CD62L^+^ or CD8^+^ CD44^+^ CD62L^−^ cells. All data are presented as Means ± SEM. Statistical significance was determined with repeated measures two-way ANOVA with two-way ANOVA with Fisher’s Least Significant Difference multiple comparisons test. N and ages are listed in materials and methods under: **a-f** Experiment 3: young isotype *N = *3, young aCD3 *N = *7, old isotype *N = *4, old aCD3 *N = *10

Memory cells can be further defined by their expression of CD62L. Central memory (CD44^+^ CD62L^+^) T cells (Tcm) primarily reside in lymphoid tissues and have reduced effector function to maintain their pool long-term for secondary responses [36]. Effector memory (CD44^+^ CD62L^−^) T cells (Tem) are highly potent cytotoxic killers. We quantified central and effector frequency and degranulation between young and old mice. In young isotype treated mice, the majority of CD8^+^ T cells are CD44^−^ CD62L^+^ naïve T cells (Tn) (51.6%) and CD44^+^ CD62L^+^ Tcms (38.7%), and upon aCD3 stimulation there is a loss of CD44^−^ CD62L^+^ Tn and CD44^+^ CD62L^+^ Tcm and an increase in the frequency of CD44^−^ CD62L^−^ and CD44^+^ CD62L^−^ Tems (Fig. 3a and b). Whereas in the old isotype mice, the CD44^−^ CD62L^+^ Tn cells only make up 8.1% of total CD8^+^ T cells, and CD44^+^ CD62L^+^ Tcms and CD44^+^ CD62L^−^ Tems make up 52.2% and 28.0% CD8^+^ T cells, respectively (Fig. 3a). When assessing CD44^+^ CD62L^±^ cells within the CD8^+^ T cell pool, old aCD3 stimulated mice have the highest proportion of CD62L^−^ Tems compared to all other groups (Fig. 3c).

We hypothesized that the difference in degranulation observed in old aCD3-treated mice may be due to different proportions of Tcm/Tem within the CD44^+^ pool. Therefore, we analyzed the expression of CD107a/CD107b within Tcm and Tem in young and old mice treated with aCD3. We observed a significant increase in CD107a/CD107b^+^ frequency and CD107a/CD107b gMFI in CD8^+^ CD44^+^ CD62L^−^ Tems compared to CD8^+^ CD44^+^ CD62L^+^ Tcms in young, but not old, aCD3 stimulated mice (Fig. 3d-f). However, there is a significant increase in CD107a/CD107b gMFI with age in both the CD44^+^ CD62L^+^ and CD44^+^ CD62L^−^ subsets (Fig. 3f). Next, we quantified the activation of Tcms and Tems in young and old mice. We found a significant expansion of CD69^+^ Tcms and Tems with aCD3 stimulation in young and old mice (Supplementary Fig. 4a-c). This was reduced in CD69^+^ Tems in old aCD3 mice compared with young aCD3 mice (Supplementary Fig. 4c). We observed an increase in GzmB^+^ frequency and gMFI in CD8^+^ CD44^+^ CD62L^+^ cells with aCD3 (Supplementary Fig. 4e and f). GzmB^+^ frequency was unchanged in Tcms between young and old aCD3 stimulated mice, but there was an increase in GzmB gMFI (Supplementary Fig. 4e and f). We observed an increase in the frequency and gMFI of GzmB in Tems with aCD3 stimulation but not age (Supplementary Fig. 4g and h). Together, these data demonstrate that CD8^+^ CD44^+^ CD62L^−^ are increased in old aCD3 stimulated mice compared to young aCD3 and old isotype mice, and this effector subset has the highest CD107a/CD107b frequency and gMFI of CD8^+^ CD44^+^ T cells, despite showing lower CD69.

### CD8^+^ CD44^+^ T cells from aged mice have reduced degranulation in vivo during cohousing challenge

In this study, we have revealed that CD8^+^ T cells from old mice have the retained ability to degranulate during supraphysiological activation with aCD3 monoclonal antibody stimulation both in vitro* and *in vivo. To assess the contribution of the aging environment on cytotoxic degranulation of CD8^+^ T cells, during the classical immune response, we challenged young and old mice with numerous murine pathogens through petstore mouse cohousing (CoH) [30, 37, 38]. CoH is a model that has been used to mature the young SPF murine immune system. We have previously observed that exhausted memory CD8^+^ CD44^+^ PD1^+^ T cells from old mice have reduced granzyme B and cytotoxic capacity, leading to impaired immunity and higher mortality [6, 39]. In this study, we observed 50% mortality of all challenged old mice within 6 days, with no deaths among young mice (Supplementary Fig. 5a). Together, these data suggest either an inability to mount a sufficient immune response or an overt immune response resulting in pathology.

**Fig. 4:**
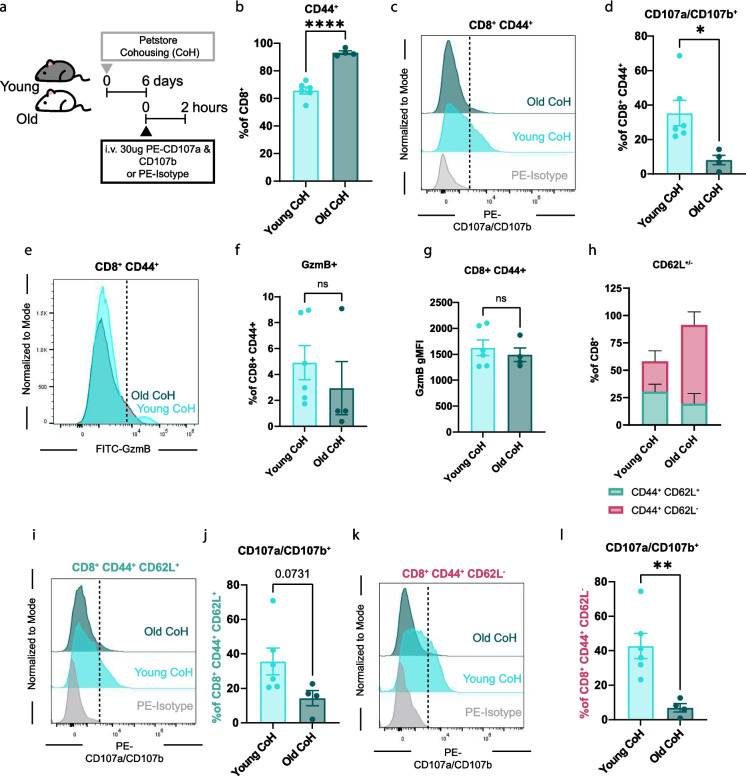
Memory CD8^+^ CD44^+^ T cells from aged mice have reduced degranulation in vivo during cohousing challenge. **a**. Diagram detailing experimental design. Young and old mice were cohoused with a petstore mouse for 6 days. On day 6 of cohousing, mice were i.v. injected with 30ug PE-CD107a and 30ug PE-CD107b or PE-Isotype control, after 2 h mice were euthanized and tissues were processed for flow cytometry analysis. **b**. Quantification of CD44^+^ cells as a frequency of CD8^+^ cells. **c.** Concatenated histograms of CD107a/CD107b expression on CD8^+^ CD44^+^ cells. The dotted line represents the positive vs negative expression of CD107a/CD107b. **d.** Quantification of CD107a/CD107b^+^ cells as a frequency of CD8^+^ CD44^+^ cells. **e.** Concatenated histograms of GzmB expression on CD8^+^ CD44^+^ cells. The dotted line represents GzmB^+^ gate. **f.** Quantification of GzmB^+^ cells as a frequency of CD8^+^ CD44^+^ cells. **g.** Quantification of GzmB gMFI on CD8^+^ CD44^+^ cells. **h.** Quantification of CD44^+^ CD62L^±^ cell populations as a frequency of CD8^+^ T cells. **i.** Concatenated histograms of CD107a/CD107b expression on CD8^+^ CD44^+^ CD62L^+^ cells. The dotted line represents CD107a/CD107b^+^ gate. The dotted line represents the positive vs negative expression of CD107a/CD107b. **j.** Quantification of CD107a/CD107b^+^ cells as a frequency of CD8^+^ CD44^+^ CD62L^+^ cells. **k.** Concatenated histograms of CD107a/CD107b expression on CD8^+^ CD44^+^ CD62L^−^ cells. The dotted line represents the positive vs negative expression of CD107a/CD107b.**l.** Quantification of CD107a/CD107b^+^ cells as a frequency of CD8^+^ CD44^+^ CD62L^−^ cells All data are presented as Means ± SEM. Statistical significance was determined with unpaired two-sided t-test with 95% confidence. N and ages are listed in materials and methods under: **a-l** Experiment 4: young CoH *N = *6, old CoH *N = *4

To assess CD8^+^ T cell degranulation, young and old mice were cohoused with a petstore mouse for 6 days, when the peak CD8^+^ T cell response occurs. On day 6 of the challenge, mice were injected with 30ug of PE-CD107a and 30ug of PE-CD107b or PE-isotype controls for 2 h to detect active degranulation of bulk CD8^+^ T cells (Fig. 4a). We first quantified whether there are differences in CD8^+^ CD44^+^ T cells during CoH between young and old mice. The frequency of CD8^+^ CD44^+^ T cells was significantly expanded in old CoH mice compared to young CoH, consistent with our previous report [6] (Fig. 4b). Next, we quantified the in vivo degranulation of young and old CD8^+^ CD44^+^ T cells via CD107a/CD107b^+^ frequency. We observed a significant decrease in CD8^+^ CD44^+^ CD107a/CD107b^+^ cells in old CoH mice compared to young CoH mice (Fig. 4c and d). We found no difference in CD8^+^ CD44^−^ CD107a/b^+^ frequency between young and old CoH mice, with the expression averaging less than 1% of CD44^−^ cells, indicating no degranulation in naïve T cells (Supplementary Fig. 5b and c). We next assessed GzmB frequency and gMFI on CD8^+^ CD44^+^ T cells to better understand the cytotoxic capacity and activation status of CD8^+^ CD44^+^ T cells in young and old mice during CoH. We found that young and old COH mice have equivalent GzmB frequency and gMFI in CD8^+^ CD44^+^ T cells (Fig. 4e-g).

We next quantified CD8^+^ subpopulations by evaluating CD44 vs CD62L expression. We observed a significant increase in CD44^+^ CD62L^−^ cells in old CoH mice compared to young CoH mice, becoming the biggest proportion of CD8^+^ T cells in old CoH mice (Fig. 4h; Supplementary Fig. 5d). We next evaluated CD107a/CD107b^+^ frequency in CD44^+^ CD62L^−/+^ cells. We found an increase in CD107a/CD107b expression above PE-isotype control-treated mice (Fig. 4i). There was no significant difference in CD8^+^ CD44^+^ CD62L^+^ CD107a/CD107b^+^ frequency between young and old CoH mice (Fig. 4i and j). However, consistent with total CD8^+^ CD44^+^ degranulation in Fig. 4D, CD44^+^ CD62L^−^ CD107a/CD107b^+^ frequency was significantly reduced in old CoH mice compared to young CoH mice (Fig. 4k and l). In both CD8^+^ CD44^+^ CD62L^+^ and CD8^+^ CD44^+^ CD62L^−^, there was a trend or significantly reduced CD107a/CD107b^+^ frequency in old CoH compared to young CoH mice (Fig. 4j and l). We observed no differences in GzmB frequency or gMFI between young CoH and old CoH mice within both the CD8^+^ CD44^+^ CD62L^+^ and CD8^+^ CD44^+^ CD62L^−^ subsets (Supplementary Fig. 5e-j). Together, these data reveal that during CoH infection, CD8^+^ T cells in old mice have reduced degranulation compared to young CoH mice despite equivalent expression of GzmB.

### Tetramer^+^ CD8^+^ T cells from aged mice have reduced degranulation in vivo during LCMV-Armstrong infection

**Fig. 5:**
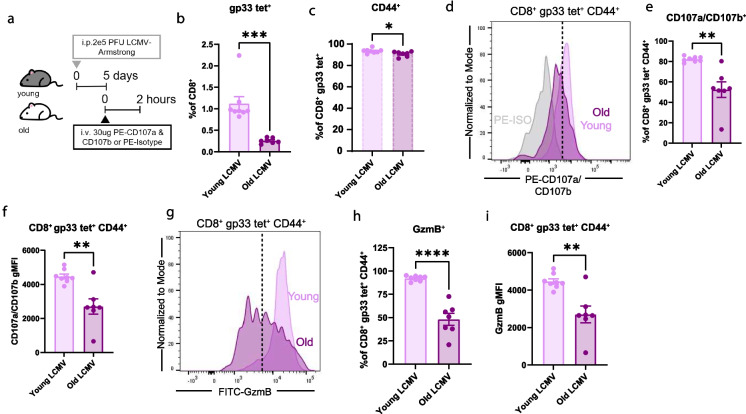
Tetramer^+^ CD8^+^ T cells from aged mice have reduced degranulation in vivo during LCMV-Armstrong infection. **a**. Diagram detailing experimental design. Young and old mice were infected with 2e5 PFU of LCMV-Armstrong for 5 days. On day 5 of infection, mice were i.v. injected with 30ug PE-CD107a and 30ug PE-CD107b or PE-isotype control. After 2 h, mice were euthanized, and tissues were processed for flow cytometry analysis. **b**. Quantification of gp33 tetramer^+^ cells as a percentage of CD8^+^ T cells. **c**. Quantification of CD44^+^ cells as a percentage of CD8^+^ gp33 tetramer^+^ cells. **d**. Concatenated histograms of CD107a/CD107b expression on CD8^+^ gp33 tetramer^+^ CD44^+^ cells. The dotted line represents CD107a/CD107b^+^ gate. **e**. Quantification of CD107a/CD107b^+^ cells as a percentage of CD8^+^ gp33 tetramer^+^ CD44^+^ cells. **f**. Quantification of CD107a/CD107b gMFI on CD8^+^ gp33 tetramer^+^ CD44^+^ cells. **g**. Concatenated histograms of GzmB expression on CD8^+^ gp33 tetramer^+^ CD44^+^ cells. The dotted line represents GzmB^+^ gate. **h**. Quantification of GzmB^+^ cells as a percentage of CD8^+^ gp33 tetramer^+^ CD44^+^ cells. **i**. Quantification of GzmB gMFI on CD8^+^ gp33 tetramer^+^ CD44^+^ cells. All data are presented as Means ± SEM. Statistical significance was determined with unpaired two-sided t-test with 95% confidence. N and ages are listed in materials and methods under: **a-i** Experiment 5: young LCMV *N = *8, old LCMV *N = *7

Next, we sought to assess the degranulation capacity, via CD107a/CD107b labeling, of antigen-specific CD8^+^ T cells from old mice. Therefore, we adopted LCMV-Armstrong as an acute model of infection. Young and old mice were infected with 2 × 10^5^ PFU of LCMV-Armstrong via i.p. injection. On day 5 of infection, mice were i.v. injected with 30ug PE-CD107a and PE-CD107b (Fig. 5a). We observed no lethality from LCMV-Armstrong in old mice within 5 days of infection. We next wanted to determine the frequency of antigen-specific cells between the young and old LCMV-infected mice; therefore, we stained cells with APC-conjugated gp33 tetramer and gated on positive cells (gp33 tet^+^). On day 5 of infection, 1.1% and 0.26% of total CD8^+^ were gp33 tet^+^ in the young LCMV and old LCMV mice, respectively (Fig. 5b;Supplementary Fig. 6a). There was a significant reduction in the frequency of CD8^+^ gp33 tet^+^ cells in the old LCMV mice compared to the young LCMV mice (Fig. 5b). Over 90% of the gp33^+^ cells were CD44^+^ (Fig. 5c). Together, these data indicate that old mice have reduced ability to generate a robust antigen-specific cell pool by day 5 of LCMV-Armstrong infection.

Despite there being fewer antigen-specific CD8^+^ cells generated in the old mice, it is unclear whether they are functionally equivalent to cells from young mice. Therefore, we measured cytotoxic capacity of CD8^+^ gp33^+^ CD44^+^ cells via degranulation (CD107a/CD107b labeling) and GzmB expression. There was an increase in CD107a/CD107b expression in young and old mice compared to PE-isotype control (Fig. 5d). The frequency and gMFI of CD107a/CD107b and GzmB were significantly reduced in CD8^+^ gp33 tet^+^ CD44^+^ cells from old LCMV-infected mice compared to young infected mice (Fig. 5d-i).

Next, we assessed the frequency of CD44 vs CD62L expression on tetramer^+^ CD8^+^ T cells from young and old LCMV mice. We observed a significant reduction in CD44^+^ CD62L^−^ cells, but an increase in all other subpopulations, as a percentage of CD8^+^ gp33 tet^+^ cells in old compared to young LCMV mice (Supplementary Fig. 6b). The CD44^+^ CD62L^−^ subpopulation made up the largest proportion of antigen-specific CD8^+^ T cells in both the young and old mice (78.8% and 60.7%, respectively) (Supplementary Fig. 6c). In both the gp33 tet^+^ CD44^+^ CD62L^+^ and gp33 tet^+^ CD44^+^ CD62L^−^ cells, there was a reduction in CD107a/CD107b and GzmB frequency and gMFI with age (Supplementary Fig. 6d-g). Together, these results indicate that old mice infected with LCMV generate reduced antigen-specific CD8^+^ T cells compared to young mice and that the cells in the old mice have reduced cytotoxic capacity.

## Discussion

The cytotoxicity of CD8^+^ T cells from older individuals is not well understood, with opposing reports revealing both increases and decreases in CD8^+^ T cell cytotoxic function through measuring target cell killing and expression of cytotoxic molecules in old organisms. In vivo, CD8^+^ T cells from aged individuals have reduced cytotoxic capacity, typically measured through granzyme and perforin expression [6, 23, 40, 41]. However, whether the dysfunction of CD8^+^ T cells from aged individuals is cell-intrinsic or due to the altered aging microenvironment (cell-extrinsic) is an important question. Therefore, we leveraged a model in which active degranulation can be measured by labeling cell surface exposure of CD107a and CD107b with fluorescent antibodies [28, 29, 32, 42]. We used this technique in vitro and in vivo, and in young and old mice. We reveal that we can detect degranulation in CD8^+^ CD44^+^ T cells and that age and exposure-specific conditions exist.

We quantified degranulation using two measurements of fluorescence, first by examining the total frequency of positive cells and second by examining the fluorescence intensity of a population of cells. In vitro, we identified a comparable increase in both measurements in activated CD8^+^ CD44^+^ T cells and a significant increase in cells from old mice compared to young mice (Fig. 1d-f). Additionally, the increase in CD107a/CD107b^+^ frequency and gMFI was also seen in CD8^+^ CD44^+^ CD69^+^ and CD69^−^ cells from old mice (Fig. 1g-i; Supplementary Fig. 2d-f). Consistent with other reports [25–27], these results suggest that CD8^+^ T cells from old mice maintain their ability to become activated, degranulate, and potentially kill target cells. Additionally, the age-related reduction of CD8^+^ T cell cytotoxicity may be due to the aged environment or the pool of naïve T cells that can respond to specific antigens. Next, we evaluated the capacity of CD8^+^ T cells from old mice to degranulate during supraphysiological activation with aCD3 in vivo. Activation with aCD3 in vivo removes the dynamics of antigen-presenting cell activation, MHC I peptide presentation, TCR repertoire, and cell migration. Altogether, removing these dynamics simplifies our model, allowing for understanding of direct T cell activation and cytotoxic capacity within the inflammatory aged environment. We believe that this model largely recapitulates the in vitro environment, as more than 50% of CD8^+^ CD44^+^ T cells upregulate CD69, consistent with ligation of CD3 and signaling in most cells, which is not observed during physiological infection within 3 h. We revealed increased CD107a/CD107b frequency and gMFI upon aCD3 intravenous stimulation in young and old mice (Fig. 2g-i). We identified a significant increase in CD107a/CD107b gMFI with age in both CD8^+^ CD44^+^ CD62L^+^ and CD62L^−^ cells (Fig. 3f) Additionally, CD107a/CD107b frequency and gMFI are increased in CD44^+^ CD62L^−^ cells compared to CD44^+^ CD62L^+^ cells in young mice and trending increase in old mice with p-values being 0.0512 and 0.0683 (Fig. 3d-f). From these results, it is clear that CD62L^−^ cells have an increased ability to degranulate compared to CD62L^+^ cells. Additionally, these data may partially explain the increase in CD107a/CD107b^+^ frequency in total CD44^+^ cells between young and old mice, as CD44^+^ CD62L^+^ cells make up a greater proportion in young aCD3 mice and CD44^+^ CD62L^−^ cells make up a greater proportion in old aCD3 mice (Fig. 3a-c). However, the explanation does not completely explain the increase in gMFI with age. Together, these results indicated that memory CD8^+^ T cells from old mice retain their intrinsic ability to become activated and degranulate, in vitro* and *in vivo, upon supraphysiological activation from aCD3. We identified that frequency and gMFI are effective measurements for detecting degranulating cells.

As aCD3 intravenous stimulation does not recapitulate a physiological exposure, we used the petstore cohousing model and LCMV-Armstrong to compare degranulation in young and old mice during infection. We found that during cohousing, CD8^+^ CD44^+^ T cells from young mice can express surface CD107a/CD107b (35.4%), indicating increased degranulation; however, cells from old mice fail to degranulate (Fig. 4c and d). Additionally, CD8^+^ CD44^+^ antigen-specific (gp33 tetramer^+^) cells from old mice have reduced CD107a/CD107b and GzmB expression compared to young LCMV infected mice (Fig. 5d-i) Together, these results show that when activated with aCD3, old CD8^+^ T cells maintain their ability to degranulate at day 6; conversely, during physiological infection, they lose this function in vivo.

This inability to degranulate may be due to several alterations within the aged microenvironment during physiological infection. One possibility is the dysfunction of APCs and their antigen presentation. APC function is diminished during aging, with reduced APC accumulation in the spleen, APCs with fewer MHC-peptide complexes, and decreased upregulation of CD40 and CD86 costimulatory molecule expression [43, 44]. Another contributing factor may be altered CD8^+^ T cell motility, with reduced CD49 d expression and impaired ability to upregulate αβ integrin chains after stimuli on CD8^+^ T cells from older individuals [45, 46]. This impaired motility alters the ability of CD8^+^ T cells to encounter APCs, migrate to peripheral tissues, and interact with target cells. Another potential factor is the constriction of the naïve T cell repertoire in old mice, as transfer of antigen-specific naïve CD8^+^ T cells, to increase their numbers, rescued old mice during West Nile Virus infection [41]. Together, alterations in the environment during aging may contribute to the reduced degranulation of CD8^+^ T cells in old mice during petstore mouse cohousing and LCMV-Armstrong infection.

In summary, our results highlight the utility of CD107a/CD107b staining in vivo to study CD8^+^ T cell cytotoxic capacity in old mice, whereas traditional functional analyses in vivo only indicate the expression of cytotoxic molecules and not their release. This work highlights the importance of considering methods of cytotoxicity measurement and models of infection to interpret results.

### Limitations of the study

This study does not assess the direct killing capacity of old CD8^+^ T cells in vitro. Additionally, we do not assess granule content in CD8^+^ T cells from young and old mice.

## Supplementary Information

Below is the link to the electronic supplementary material.

Supplementary file1 (PDF 3320 KB)

Supplementary file2 (PDF 28.6 KB)

## Data Availability

The data presented in the study are included in the article and the Supplementary material. Further inquiries or raw data requests can be made to the corresponding author.
