# Supplementary material for: In vivo labeling reveals that degranulation is increased under supraphysiological TCR stimulation, but not infection, in CD8+ T cells from old mice
Source: GeroScience. 2025 Jun 6;48(1):897–913. doi: 10.1007/s11357-025-01723-5 (PMC12972495; doi:10.1007/s11357-025-01723-5)
Supplement: Supplementary file 1 — Supplementary file1 (PDF 3320 KB) [file 11357_2025_1723_MOESM1_ESM.pdf]

a

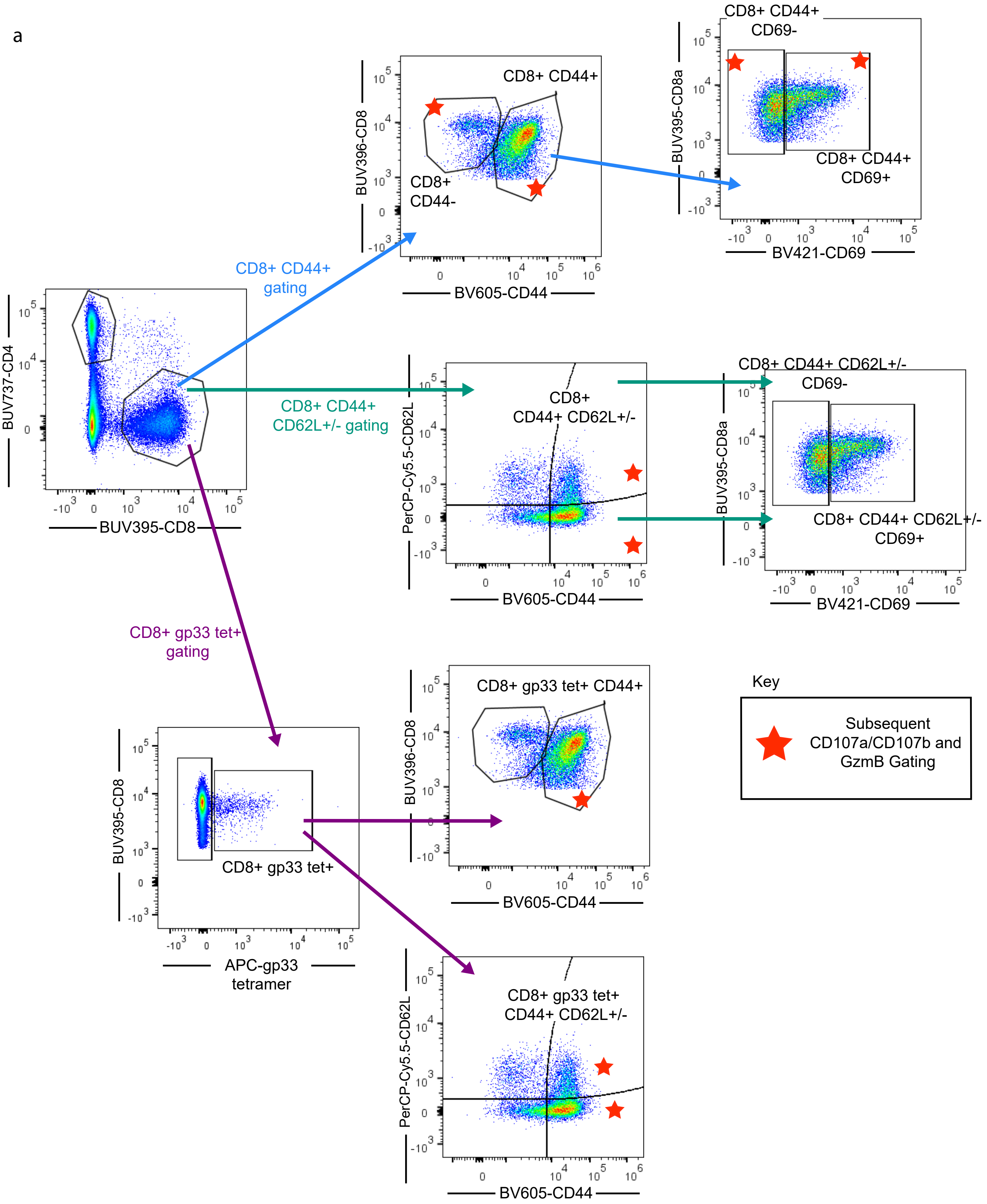

Supplementary Figure 1

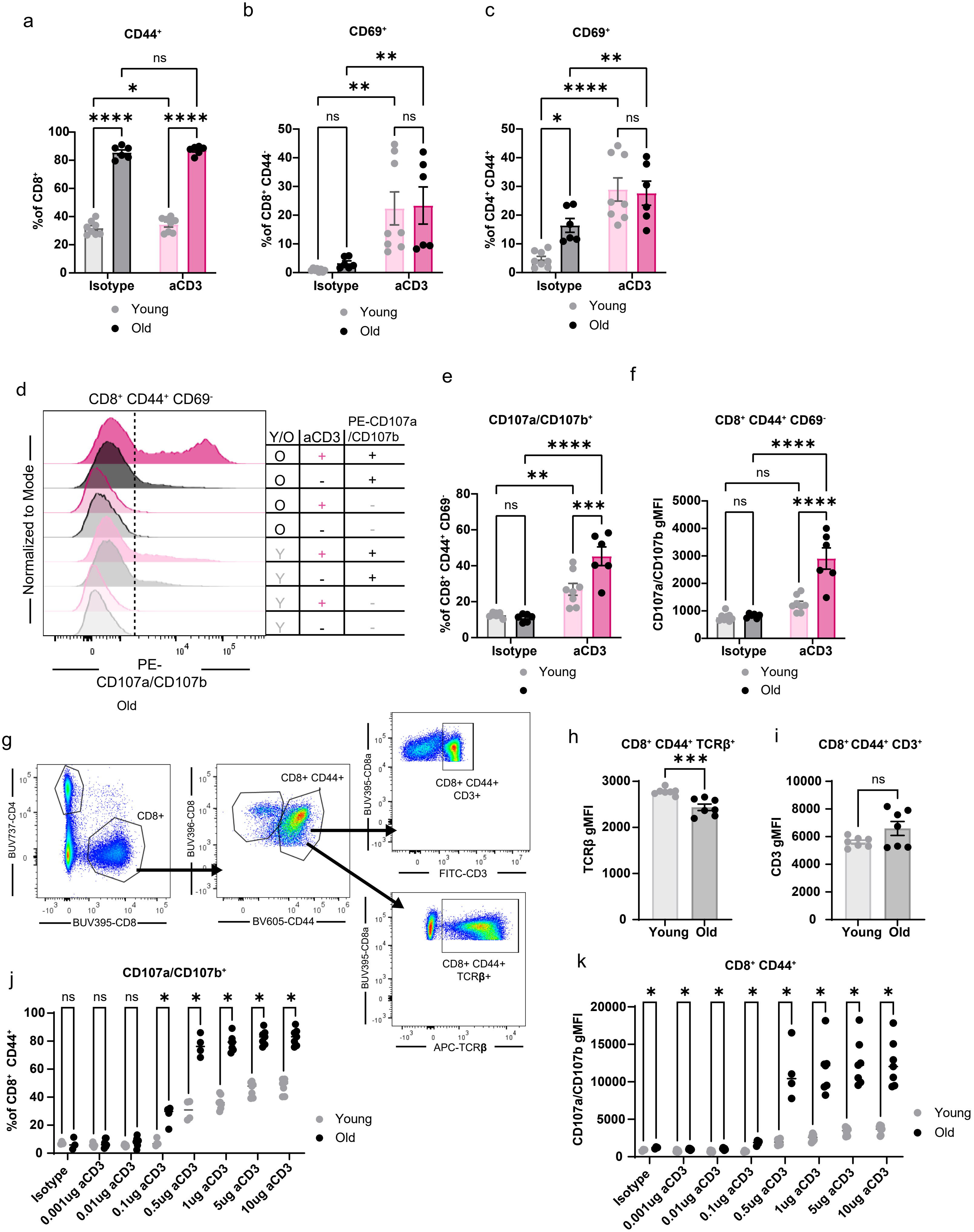

Supplementary Figure 2

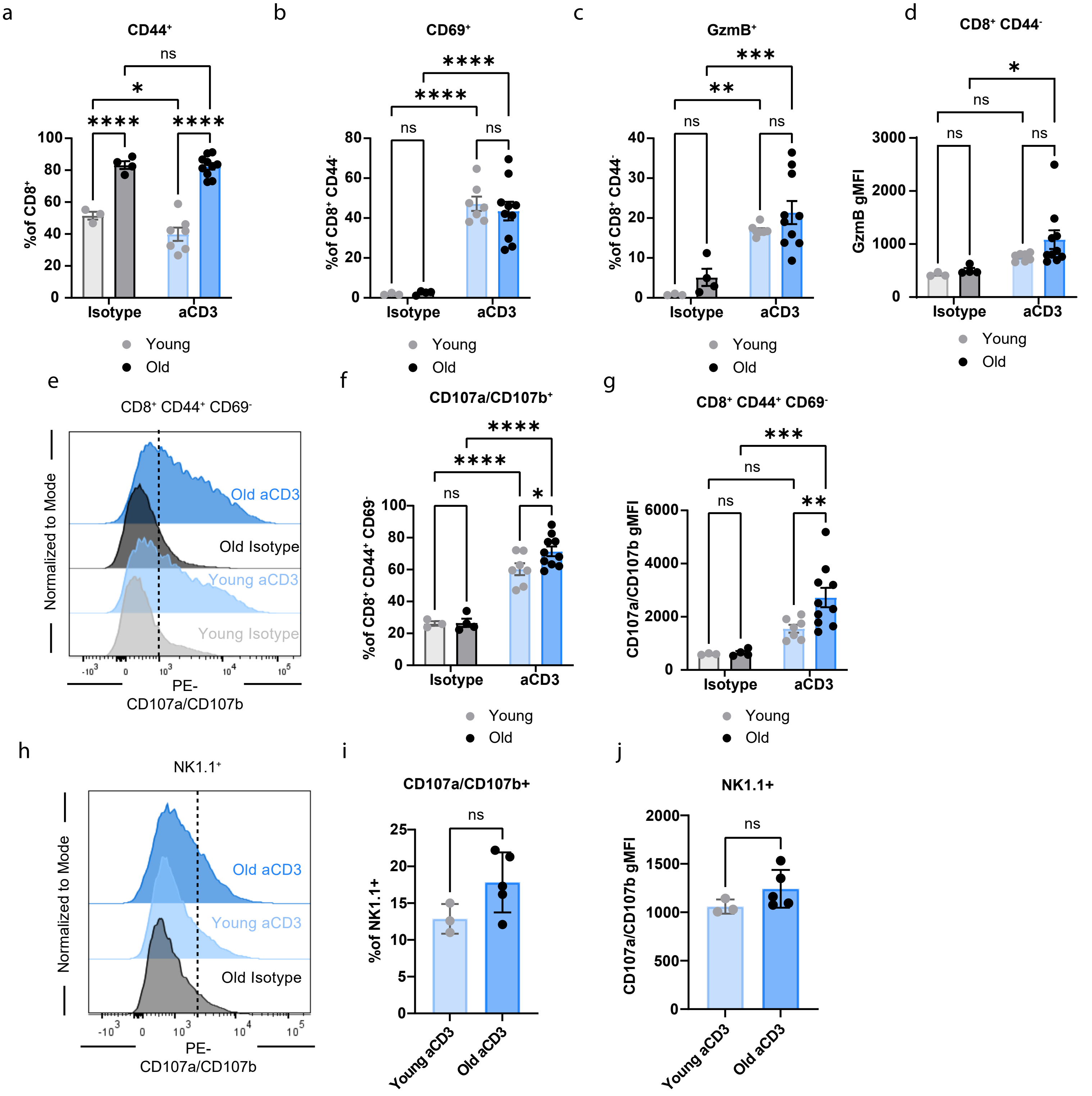

Supplementary Figure 3

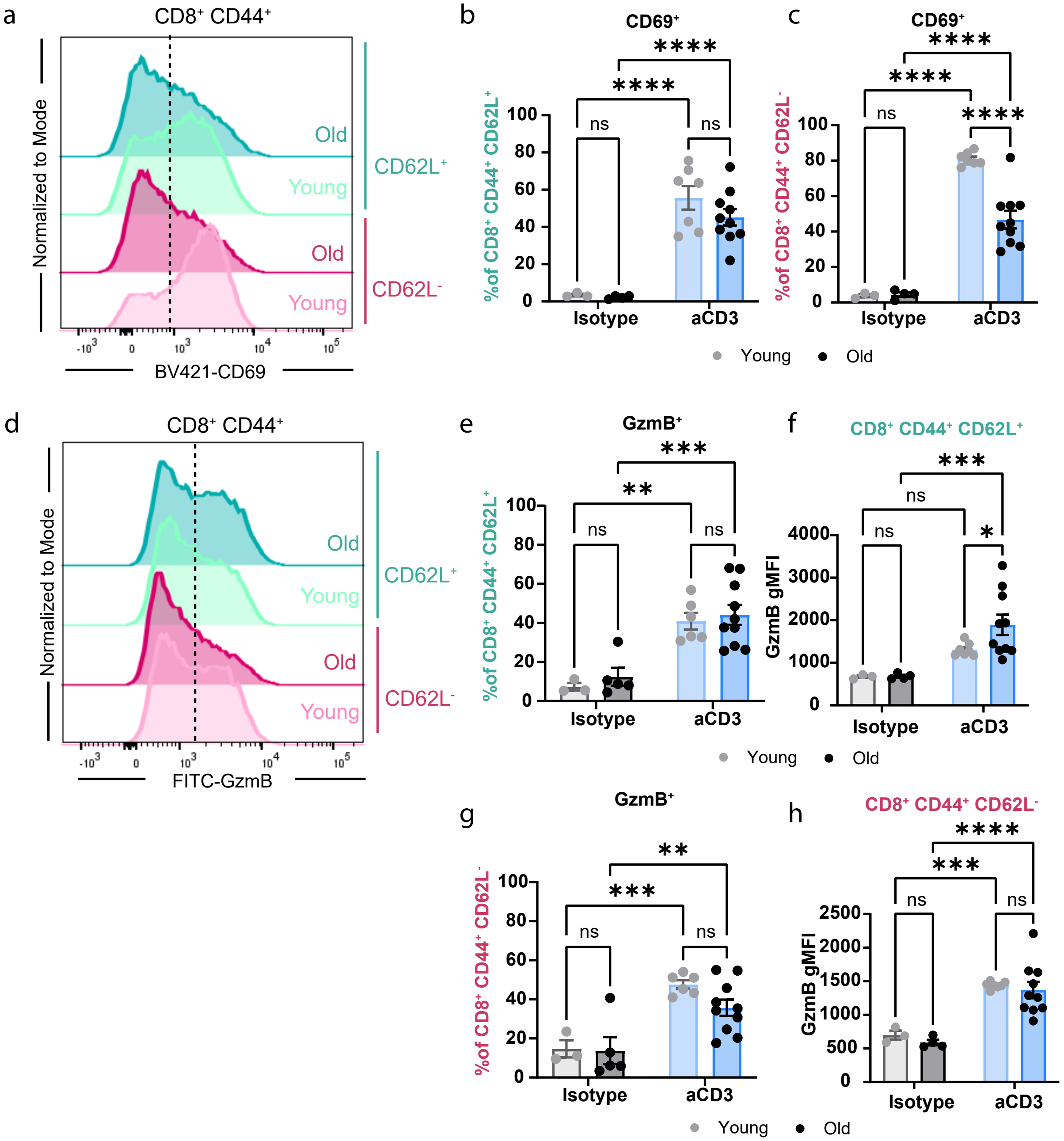

Supplementary Figure 4

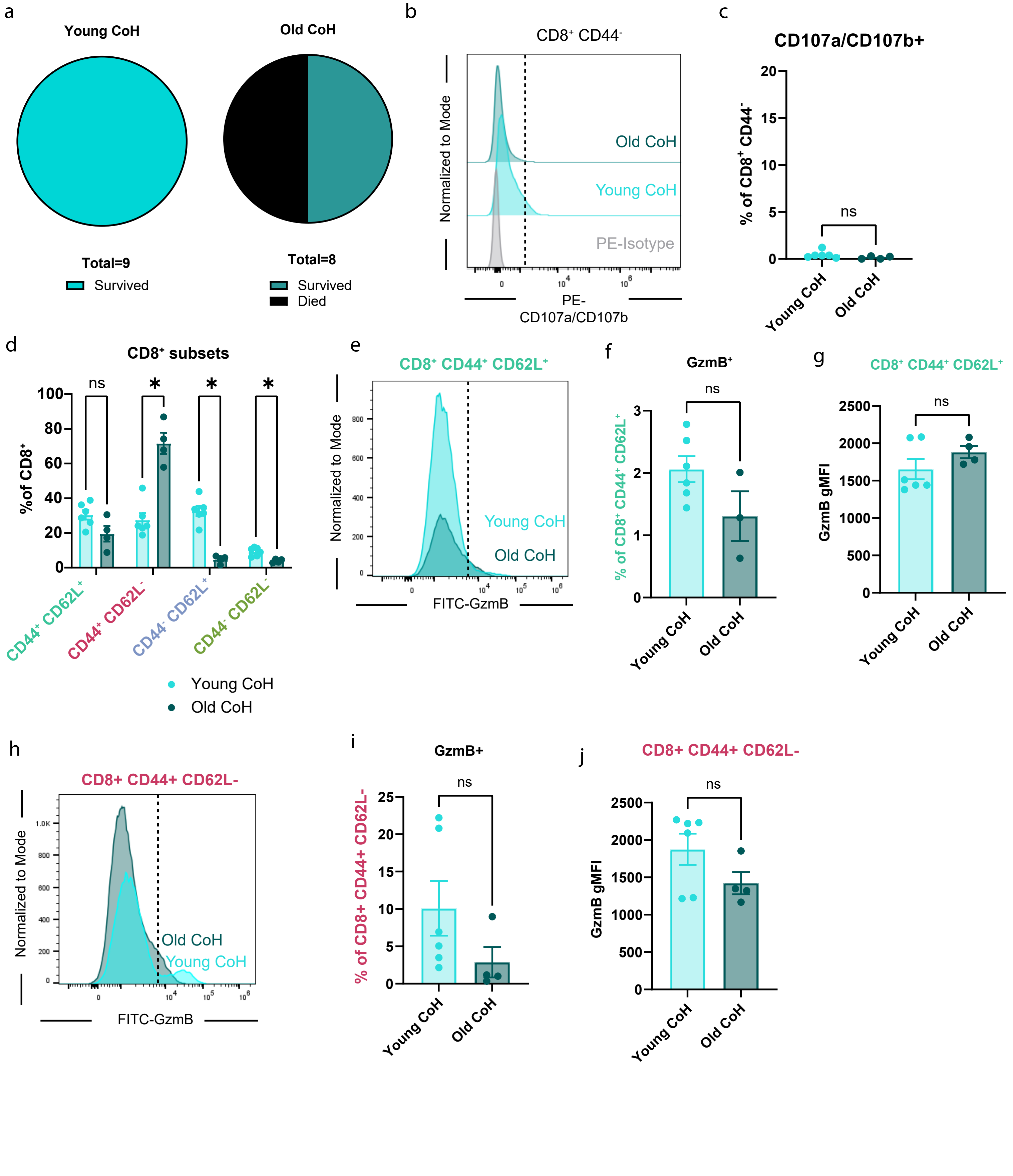

Supplementary Figure 5

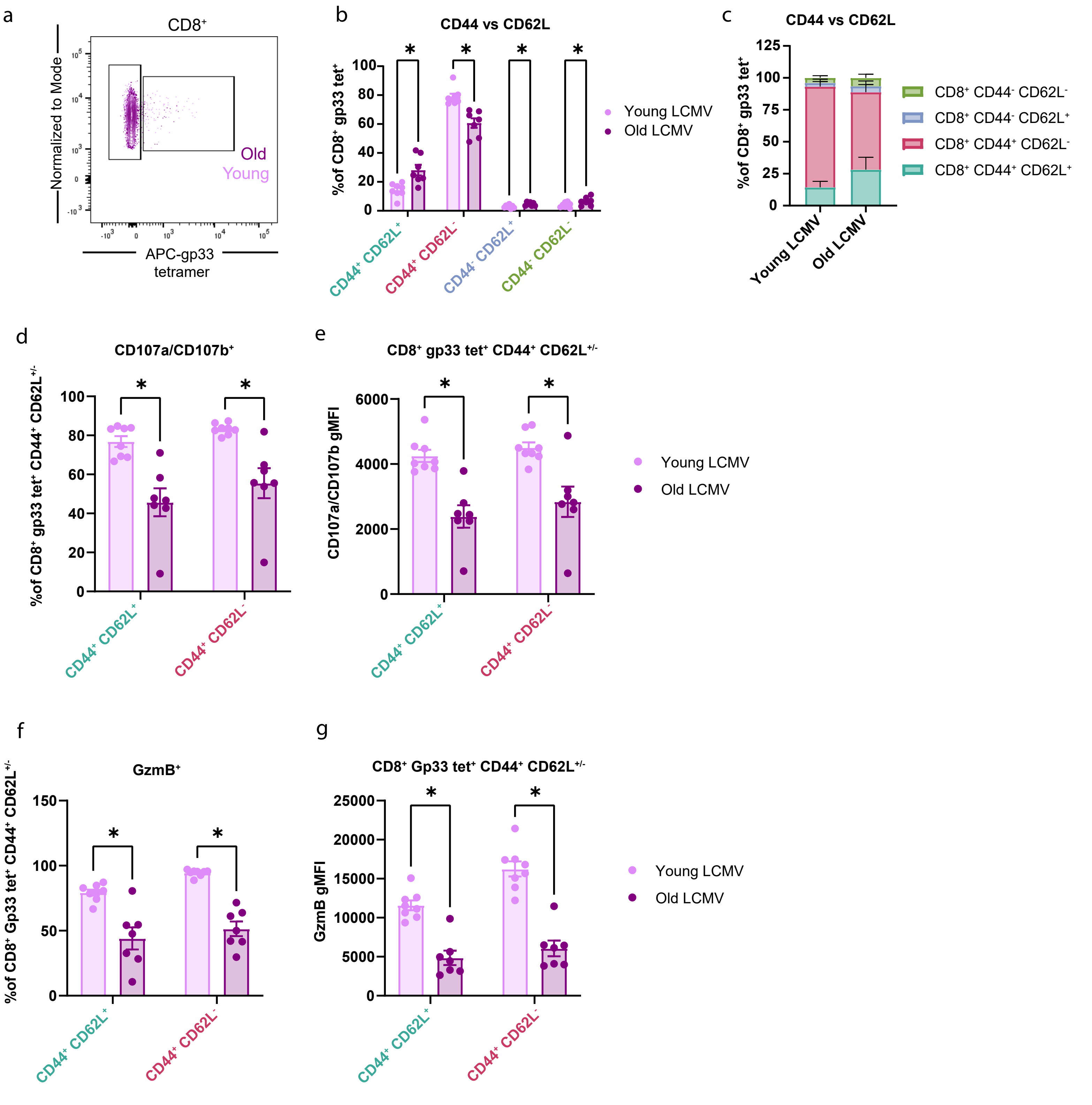

Supplementary Figure 6
