# Supplementary material for: In vivo labeling reveals that degranulation is increased under supraphysiological TCR stimulation, but not infection, in CD8+ T cells from old mice
Source: GeroScience. 2025 Jun 6;48(1):897–913. doi: 10.1007/s11357-025-01723-5 (PMC12972495; doi:10.1007/s11357-025-01723-5)
Supplement: Supplementary file 2 — Supplementary file2 (PDF 28.6 KB) [file 11357_2025_1723_MOESM2_ESM.docx]

**Supplementary Figure 1. Gating Strategy**

**a.** Pseduocolor plots detailing flow cytometry gating strategies.

**Supplementary Figure 2. Memory CD8^+^ CD44^+^ T cells from old mice have increased degranulation *in vitro*.**

**a.** Quantification of CD44^+^ cells as a percentage of CD8^+^ cells. **b.** Quantification of CD69^+^ cells as a percentage of CD8^+^ CD44^-^ cells. **c.** Quantification of CD69^+^ cells as a percentage of CD4^+^ CD44^+^ cells. **d.** Concatenated histograms of CD107a/CD107b expression on CD8^+^ CD44^+^ CD69^-^ T cells. The dotted line represents the positive vs negative expression of CD107a/CD107b. **e.** Quantification of CD107a/CD107b^+^ cells as a percentage of CD8^+^ CD44^+^ CD69^-^ cells. **f.** Quantification of CD107a/CD107b gMFI on CD8^+^ CD44^+^ CD69^-^ cells. **g.** Representative gating strategy for CD8^+^ CD44^+^ TCRβ^+^ or CD8^+^ CD44^+^ CD3^+^ cells. **h.** Quantification of TCRβ gMFI on CD8^+^ CD44^+^ TCRβ^+^ cells. **i.** Quantification of CD3 gMFI on CD8^+^ CD44^+^ CD3^+^ cells. **j.** Quantification of CD107a/CD107b^+^ cells as a percentage of CD8^+^ CD44^+^ cells after stimulation with 0.001-10µg of aCD3 antibody. **k.** Quantification of CD107a/CD107b gMFI on CD8^+^ CD44^+^ cells after stimulation with 0.001-10µg of aCD3 antibody. All data are presented as Means ± SEM. Statistical significance was determined with repeated measures two-way ANOVA with two-way ANOVA with Fisher’s Least Significant Difference multiple comparisons test. N and ages are listed in materials and methods under: (**a-f**) Experiment 1: young N=8; old N=6. (**g-k**) Experiment 2: young N=7, old N=7.

**Supplementary Figure 3. Memory CD8^+^ CD44^+^ T cells from old mice have maintained degranulation *in vivo*.**

**a.** Quantification of CD44^+^ cells as a percentage of CD8^+^ cells. **b.** Quantification of CD69^+^ cells as a percentage of CD8^+^ CD44^-^ cells. **c.** Quantification of GzmB^+^ cells as a percentage of CD8^+^ CD44^-^ cells. **d.** Quantification of GzmB gMFI on CD8^+^ CD44^-^ cells. **e.** Concatenated histograms of CD107a/CD107b expression on CD8^+^ CD44^+^ CD69^-^ T cells. The dotted line represents the positive vs negative expression of CD107a/CD107b. **f.** Quantification of CD107a/CD107b^+^ cells as a percentage of CD8^+^ CD44^+^ CD69^-^ cells. **g.** Quantification of CD107a/CD107b gMFI on CD8^+^ CD44^+^ CD69^-^ cells. **h.** Concatenated histograms of CD107a/CD107b expression on NK1.1^+^ cells. The dotted line represents the positive vs negative expression of CD107a/CD107b. **i.** Quantification of CD107a/CD107b^+^ cells as a percentage of NK1.1^+^ cells. **j.** Quantification of CD107a/CD107b gMFI on NK1.1^+^ cells. All data are presented as Means ± SEM. Statistical significance was determined with repeated measures two-way ANOVA with two-way ANOVA with Fisher’s Least Significant Difference multiple comparisons test. N and ages are listed in materials and methods under: (**a-g**) Experiment 3: young isotype N=3, young aCD3 N=7, old isotype N=4, old aCD3 N=10. (**h-j**) Experiment 3-Part 3 only: young aCD3 N=3, old aCD3 N=5.

**Supplementary Figure 4. Tem CD8^+^ CD44^+^ CD62L^-^ T cells have the highest CD107a/CD107b expression in old mice.**

**a.** Concatenated histograms of CD69 expression on CD8^+^ CD44^+^ CD62L^+^ (Tcms) and CD8^+^ CD44^+^ CD62L^-^ (Tems). The dotted line represents the CD69^+^ gate. **b.** Quantification of CD69^+^ cells as a frequency of Tcms. **c.** Quantification of CD69^+^ cells as a frequency of CD44^+^ CD62L^-^ cells. **d.** Concatenated histograms of GzmB expression on CD8^+^ CD44^+^ CD62L^+^ and CD8^+^ CD44^+^ CD62L^-^ cells. The dotted line represents the GzmB^+^ gate. **e.** Quantification of GzmB^+^ cells as a frequency of CD8^+^ CD44^+^ CD62L^+^ cells. **f.** Quantification of GzmB gMFI on CD8^+^ CD44^+^ CD62L^+^ cells. **g.** Quantification of GzmB^+^ cells as a frequency of CD8^+^ CD44^+^ CD62L^-^ cells. **h.** Quantification of GzmB gMFI on CD8^+^ CD44^+^ CD62L^-^ cells. All data are presented as Means ± SEM. Statistical significance was determined with repeated measures two-way ANOVA with two-way ANOVA with Fisher’s Least Significant Difference multiple comparisons test. N and ages are listed in materials and methods under: (**a-h**) Experiment 3: young isotype N=3, young aCD3 N=7, old isotype N=4, old aCD3 N=10.

**Supplementary Figure 5. Memory CD8^+^ CD44^+^ T cells from aged mice have reduced degranulation *in vivo* during cohousing challenge.**

**a.** Pie charts representing proportions of survival and mortality in young CoH and old CoH mice. **b.** Concatenated histograms of CD107a/CD107b expression on CD8^+^ CD44^-^ cells. Dotted line represents CD107a/CD107b^+^ gate. **c.** Quantification of CD107a/CD107b^+^ cells as a frequency of CD8^+^ CD44^-^ cells. **d.** Quantification of CD44 vs CD62L cell populations as a frequency of CD8^+^ T cells. **e.** Concatenated histograms of GzmB expression on CD8^+^ CD44^+^ CD62L^+^ cells. The dotted line represents GzmB ^+^ gate. **f.** Quantification of GzmB^+^ cells as a frequency of CD8^+^ CD44^+^ CD62L^+^ cells. **g.** Quantification of GzmB gMFI on CD8^+^ CD44^+^ CD62L^+^ cells. **h.** Concatenated histograms of GzmB expression on CD8^+^ CD44^+^ CD62L^-^ cells. The dotted line represents GzmB ^+^ gate. **i.** Quantification of GzmB^+^ cells as a frequency of CD8^+^ CD44^+^ CD62L^-^ cells. **j.** Quantification of GzmB gMFI on CD8^+^ CD44^+^ CD62L^-^ cells. All data are presented as Means ± SEM. Statistical significance was determined with unpaired two-sided t-test with 95% confidence (**c, f, g, i, j**) or multiple unpaired t-tests with 95% confidence (**d**). N and ages are listed in materials and methods under: (**a-j**) Experiment 4: young CoH N=6, old CoH N=4.

**Supplementary Figure 6. Tetramer^+^ CD8^+^ T cells from aged mice have reduced degranulation *in vivo* during LCMV-Armstrong infection.**

**a.** Concatenated contour plot of APC-gp33 tetramer^+^ young and old CD8^+^ cells. **b.** Quantification of CD44 vs CD62L populations as a percentage of CD8^+^ gp33 tetramer^+^ cells. **c.** Quantification of CD44 vs CD62L populations as a percentage of CD8^+^ gp33 tetramer^+^ cells. **d.** Quantification of CD107a/CD107b^+^ cells as a percentage of CD8^+^ gp33 tetramer^+^ CD44^+^ CD62L^+/-^ cells. **e.** Quantification of CD107a/CD107b gMFI on CD8^+^ gp33 tetramer^+^ CD44^+^ CD62L^+/-^ cells. **f.** Quantification of GzmB^+^ cells as a percentage of CD8^+^ gp33 tetramer^+^ CD44^+^ CD62L^+/-^ cells. **g.** Quantification of GzmB gMFI on CD8^+^ gp33 tetramer^+^ CD44^+^ CD62L^+/-^ cells. All data are presented as Means ± SEM. Statistical significance was determined with multiple unpaired two-sided t-test with 95% confidence. N and ages are listed in materials and methods under: (**a-i**) Experiment 5: young LCMV N=8, old LCMV N=7.
